# Motor Decline in Clinically Presymptomatic Spinocerebellar Ataxia Type 2 Gene Carriers

**DOI:** 10.1371/journal.pone.0005398

**Published:** 2009-04-29

**Authors:** Luis Velázquez-Perez, Rosalinda Díaz, Ruth Pérez-González, Nalia Canales, Roberto Rodríguez-Labrada, Jacquelín Medrano, Gilberto Sánchez, Luis Almaguer-Mederos, Cira Torres, Juan Fernandez-Ruiz

**Affiliations:** 1 Centro para la Investigación y Rehabilitación de las Ataxias Hereditarias, Holguín, Cuba; 2 Laboratorio de Neuropsicología, Departamento de Fisiología, Facultad de Medicina, Universidad Nacional Autónoma de México, Ciudad de México, Distrito Federal, México; 3 Facultad de Psicología, Universidad Veracruzana, Xalapa, Veracruzana, México; National Institutes of Health, United States of America

## Abstract

**Background:**

Motor deficits are a critical component of the clinical characteristics of patients with spinocerebellar ataxia type 2. However, there is no current information on the preclinical manifestation of those motor deficits in presymptomatic gene carriers. To further understand and characterize the onset of the clinical manifestation in this disease, we tested presymptomatic spinocerebellar ataxia type 2 gene carriers, and volunteers, in a task that evaluates their motor performance and their motor learning capabilities.

**Methods and Findings:**

28 presymptomatic spinocerebellar ataxia type 2 gene carriers and an equal number of control volunteers matched for age and gender participated in the study. Both groups were tested in a prism adaptation task known to be sensible to both motor performance and visuomotor learning deficits. Our results clearly show that although motor learning capabilities are intact, motor performance deficits are present even years before the clinical manifestation of the disease start.

**Conclusions:**

The results show a clear deficit in motor performance that can be detected years before the clinical onset of the disease. This motor performance deficit appears before any motor learning or clinical manifestations of the disease. These observations identify the performance coefficient as an objective and quantitative physiological biomarker that could be useful to assess the efficiency of different therapeutic agents.

## Introduction

Spinocerebellar ataxia type 2 (SCA2) is a genetic-based disorder with primary symptoms of progressive gait ataxia, diminished saccade velocity [1], poor coordination of speech musculature (dysarthria), olfactory deficits [2], [3] and absence of neurological reflexes such as the knee jerk reaction (areflexia) [4]–[6]. This polyglutamine (PolyQ) disorder produces severe degeneration of pontine nuclei, inferior olives, and Purkinje cells in the cerebellum [7], [8].

A general characteristic of hereditary neurodegenerative diseases is that clinical symptoms not necessarily emerge from birth, but can appear throughout different life stages. The specific age at which overt clinical manifestations appear is in close relationship to the individual repeat length [9]. Nevertheless, different studies have demonstrated that in several neurodegenerative diseases, specific deficiencies can be detected in presymptomatic gene carrier individuals. For example, deficits has been found in clinically presymptomatic familial autosomic dominant Alzheimer [10], or in clinically presymptomatic Huntington gene carriers individuals, who show deficits in motor control [11]–[14], learning of motor sequences [15], and other non-motor functions, like memory and executive functions [16]–[18], visuospatial processing [19] and olfaction [20]. These kinds of deficits are usually detected only through specialized testing.

Early symptoms reported by SCA2 patients appearing before gait ataxia, which is the most notorious clinical deficit, include dysarthria, problems with hand writing, and sleep disturbances [21]. However, to our knowledge (based on public Medline and scholar Google searches) there are only two studies that have found preclinical deficits in SCA2 gene carriers. Those reports show brisk deep-tendon reflexes [22] and electrophysiological alterations in sensory nerve conduction, and somatosensory and auditory evoked potentials [23].

To understand the progressive changes taking place before the onset of the clinical manifestations in SCA2, we evaluated the performance and visuomotor learning capabilities of presymptomatic gene carrier individuals. For this purpose, we used a task sensible to both, SCA2 gene mutation [24] and cerebellar damage [25].

## Methods

### Subjects

Twenty eight clinically presymptomatic SCA2 gene carriers (11 male and 17 female patients) with ages ranging from 27 to 63 years (mean, 39.6; standard deviation (S.D)±9.1), probable age of onset from 28 to 63 years (mean, 45; S.D.±2), and polyglutamine repeat sizes from 32 to 41 repetitions (mean, 36.6; S.D.±2.6) were admitted to the Center for the Research and Rehabilitation of Hereditary Ataxias in Holguín for this study (see Table 1 for individual demographic information). The diagnosis of SCA2 was based on genealogical descent from the founder population, and on molecular genetic determination of the repeat expansions. The clinical assessment was conducted using a standard neurological exam [26] and the Scale for the assessment and rating of ataxia (SARA) [27]. This scale comprise eight items: evaluation of gait (score 0–8), stance (score 0–6), sitting (score 0–4) and speech (score 0–6), and four tests assessing limb kinetic function (finger chase [score 0–4], fast alternating hand movements [score 0–4], finger-nose-finger test [score 0–4] and heel-shin test [score 0–4]) evaluates the core clinical symptoms in ataxia diseases [27].

**Table 1:** Presymptomatic SCA2 gene carriers demographic information.

Ss	Age	Gender	Lineage	Normal allele repetitions	Mutated allele repetitions	Predicted AACO	Years to AACO	SARA score
1	41	f	Paternal	22	36	44.26	3.00	0
2	31	f	Paternal	22	32	63.70	32.70	1
3	63	f	Maternal	22	34	53.10	−9.90	0
4	61	f	Maternal	22	34	53.10	−7.90	0
5	27	f	Maternal	22	35	30.76	3.76	0
6	47	f	Paternal	22	32	63.70	16.70	0
7	34	f	Paternal	22	34	53.10	19.10	0
8	31	f	Paternal	24	39	33.69	2.69	0
9	38	f	Maternal	29	34	33.69	−4.31	0
10	37	f	Paternal	29	37	40.41	3.41	0
11	44	f	Maternal	24	39	48.48	4.48	0
12	32	f	Paternal	22	38	36.90	4.90	1
13	40	f	Paternal	34	39	53.10	13.10	0
14	32	f	Paternal	22	33	58.15	26.15	0
15	37	f	Maternal	22	36	44.26	7.26	1
16	42	f	Paternal	22	38	36.90	−5.10	0
17	39	f	Paternal	22	35	48.48	9.48	0
18	35	m	Paternal	22	35	48.48	13.48	0
19	39	m	Maternal	23	38	48.48	9.48	0
20	41	m	Maternal	22	36	44.26	3.26	1
21	41	m	Maternal	24	38	36.90	−4.10	0
22	35	m	Paternal	22	36	44.26	9.26	0
23	34	m	Maternal	22	41	28.08	−5.92	0
24	50	m	Paternal	22	35	48.48	−1.52	1
25	58	m	Maternal	22	34	53.10	−4.90	0
26	32	m	Paternal	22	36	44.26	12.26	0
27	41	m	Maternal	22	41	28.08	−12.92	0
28	28	m	Paternal	23	41	28.08	0.08	2

AACO is the probable age at clinical onset. SARA is the Scale for the assessment and rating of ataxia.

The predicted age at clinical onset (AACO) correlating to each polyglutamine expansion size was calculated after the formula originally published in the article describing the SCA2 mutation: [AACO] = 1171.583 * e ^(−0.091* polyglutamine expantion size)^ [9]. A group of 28 age and gender-matched unpaid healthy adult volunteers with no history of neurological injury or psychiatry disease from Holguín province (11 male and 17 female subjects) with ages ranging from 27 to 61 years (means, 38; S.D.±9.6) served as controls (CON). The control group also had the same clinical assessment and SARA evaluations as the SCA2 patients. Subjects with previous history of any other major disorder that could affect visuomotor performance were not entered into the study. All SCA2 patients and controls were right handed. The study plan was approved by the Center for the Research and Rehabilitation of Hereditary Ataxias in Holguín and National Autonomous University of Mexico ethics committees before recruitment of participants started. Therefore, all the experimentation procedures followed were in accordance with the ethical standards of both committees. In addition, all subjects signed an informed consent prior to the experiments in accordance with the Helsinki Declaration (Council for International Organizations of Medical Sciences and World Health Organization, 2002).

### Behavioral task

A detailed description of the procedure and our modifications can be found elsewhere [25], [28]. Subjects threw clay balls (weight: 10 g) at a 12-cm×12-cm cross drawn on a large sheet of parcel paper centered at shoulder level and placed 2 m away in front of them. Subjects were instructed to make each toss overhand during the whole experiment, to use the right hand, and to throw the balls to the location where they saw the target. The subjects had an unobstructed view of the target during the entire session. Subjects stood, the head was unrestrained, and no directions were given about trunk, shoulder or head/neck posture [25]. However, they were not allowed to look down at their hand as they collected the next ball from a tray located next to them. Subjects were asked to throw at their own pace, so they were free to take rests if they felt tired. If such event occurred, they were asked to remain still as possible with their eyes closed.

The experiment had three conditions. Under each condition the subjects threw 26 balls. During the baseline condition (PRE) subjects did not wear prisms. After finishing the baseline condition subjects were tested in the PRISM condition, where they wore 30 diopter Fresnel 3 M Press-on plastic lenses (3 M Health Care, Specialties Division, St. Paul, MN, USA) that produce a light refraction to the right. Once that condition was finished, subjects had the prisms removed and started the POS condition where they continued throwing balls. The location of the balls impacts were plotted sequentially by trial number (abscissa) versus horizontal displacement (in centimeters) from a vertical line passing through the target centre (ordinate). Impacts to the left of the target were plotted as negative values and impacts to the right were plotted as positive values. The three experimental conditions were carried out consecutively after the donning or doffing of the prisms was completed.

Three additional measures were calculated from the collected data of both experiments. First, a motor performance coefficient (PC) (or called variable errors [24], [25]) was calculated from the baseline phase. To obtain the PC, the horizontal errors (distance from each impact location to a vertical line passing through the target) of the PRE trials were measured. The PC is the standard deviation obtained from these errors [25]. Second, an adaptation magnitude was obtained by subtracting the horizontal distance to the target on the final throw from that on the initial throw while wearing the prisms (PRISM condition). Third, an aftereffect measure was defined as the ball's impact horizontal distance to the target on the first throw after removing the prisms.

## Results

### Predicted time to clinical manifestations

Time to clinical manifestation was calculated as years remaining from subjects' current age at the time of testing to the calculated age of onset accordingly to their polyglutamine expansion size. The analysis suggested that the mean time to start clinical manifestations for this specific population of presymptomatic subjects was 5.97±2.21 years.

### Ataxia score

All the pre-SCA2 gene carriers had a SARA score between 0 and 2 (mean 0.23±0.49 SDM), of the possible maximum score of 40. Since previous studies have found normal subjects showing scores as high as 7, all the pre-SCA2 participants could not be differentiated from normal volunteers based on the SARA score, confirming their clinically presymptomatic status [27]. In the present study all the control volunteers had a score between 0 and 0.5, so they did not show any deficit measured with the SARA.

### Motor performance coefficient

**Figure 1:**
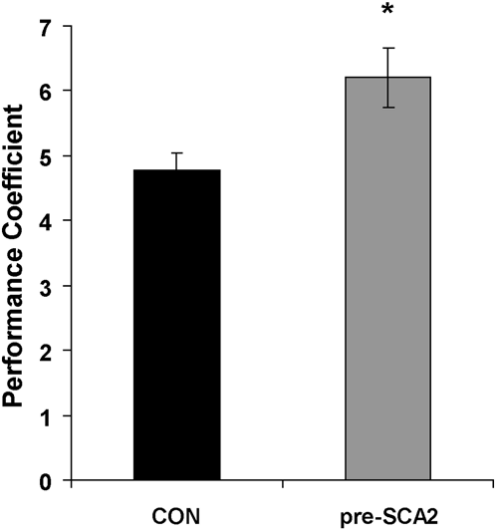
Motor performance coefficient of the control (black) and the pre-SCA2 (grey) groups. Error bars are SEM. * = p<0.01.

Measurements from the throws made during the baseline condition without visual perturbation were used to evaluate motor performance. Motor performance coefficients for each group were as follows: CON = 4.77±0.26 s.e.m., and pre-SCA2 = 6.2±0.45 s.e.m. (Figure 1). A Kolmogorov-Smirnov normality test shows that although the data distribution for the control group passed (p = 0.066) the test, the pre-SCA 2 group failed it (p<0.05). Therefore a Mann-Whitney rank sum test was used to compare between both groups. The results showed a significant difference between controls and patients (U = 541, T = 649, n = 28, p = 0.01).

### Adaptation

**Figure 2:**
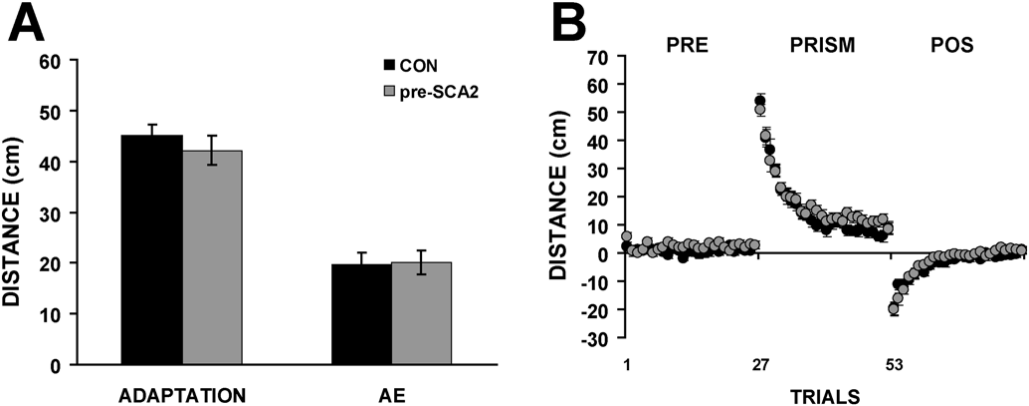
Adaptation, aftereffect, and trial-by-trial distance to target. (A) Adaptation (left) and aftereffect (AE) (right) distance measures for the control (black) and the pre-SCA2 (grey) groups. (B) Trial-by-trial distance to target in PRE, PRISM, and POS conditions for the control (black) and the pre-SCA2 (grey) groups. Error bars are SEM. Note the lack of differences between groups in A and B.

The adaptation measures obtained during the prism phase were as follows: CON = 44.9±2.2, and preSCA2 = 42.1±2.8 (Figure 2 Left). A Kolmogorov-Smirnov normality test showed that both groups data passed the normality (p = 0.794) and equal variance tests (p = 0.195). A two-tailed Student's t test was performed to look for adaptation differences between control subjects and presymptomatic gene SCA2 carriers. The analysis showed no differences between groups (t = 0.7, d.f. = 54, p = 0.43) (Figure 2A).

To analyze de adaptation rate, a two way repeated measures ANOVA was used. The conditions were groups (Controls and pre-SCA2) and throws. The analysis showed that there were no differences between both groups (d.f. = 1, F = 1.156, p = 0.287), but, as expected, there were significant differences among throws (d.f. = 25, F 77.213, p<0.01). However, the analysis also showed that there were no significant interactions between groups and throws (d,f, = 25, F = 1.151, p = 0.276) (Figure 2B).

### Aftereffects

The aftereffect absolute values were as follows: CON = 19.6±2.2 s.e.m., preSCA2 = 20±2.2 s.e.m. (Figure 2A). A Kolmogorov-Smirnov normality test showed that both groups data passed the normality (p = 0.270) and equal variance tests (p = 0.874). A two-tailed Student's t test was performed for analyzing possible aftereffect differences between control and presymptomatic gene SCA2 carriers. The analysis showed no differences between groups (t = 0.14, d.f. = 54, p = 0.88).

### The motor deficit and time to start clinical manifestations in SCA2 gene carriers

Since the experimental results show motor deficits in the clinical presymptomatic SCA2 gene carriers, an important question was if there is a correlation between those deficits and the years remaining to reach the predicted age at clinical onset. A Pearson product moment correlation showed a significant correlation between those two variables (CC = 0.437, p = 0.02).

## Discussion

The present results suggest that clinically presymptomatic SCA2 gene carriers show an early motor deficit manifested as deterioration in their motor performance coefficient. This deficit can be detected on average even five years before the clinical manifestations onset. The neural deterioration causing the motor deficit is not enough, however, to disrupt neither their motor learning nor the measures obtained from the SARA scale.

The motor performance deficit in presymptomatic individuals could be explained if the neurodegeneration starts years before the clinical onset. For example, it has been established that SCA2 asymptomatic individuals show cerebellar hypometabolism and fourth ventricle dilatation even years before clinical manifestation of the disease [29]. Hence, it would be interesting to test if the motor performance deficit could be evidence of an incipient neurodegeneration, since it shows a positive correlation with the years remaining to reach the predicted age at clinical onset.

The task used here can measure both, motor performance and visuomotor learning. Initial findings suggest that these two variables are independent, since they can be dissociated in patients with different cerebellar lesions [25]. Therefore, it could be possible that SCA2 initially affects structures important for motor performance but not motor learning. On the other hand, compensatory mechanisms have been found during the course of neurodegenerative diseases, as suggested by activity changes found in regions not necessarily damaged in the course of the disease [15], [30]. This evidence opens the possibility that these mechanisms could mask or protect processes or structures involved in motor learning, but not necessarily those involved in motor performance.

In conclusion, the current findings show a clear deficit in motor performance that can be detected years before the clinical onset of the disease. This motor performance deficit appears before any motor learning or clinical manifestations of the disease. These observations identify PC as an objective and quantitative physiological biomarker that could be of potential use to assess the efficiency of different therapeutic agents.
